# Supplementary material for: Curated and harmonised transcriptomics datasets of interstitial lung diseases
Source: Data Brief. 2025 Oct 14;63:112139. doi: 10.1016/j.dib.2025.112139 (PMC12581653; doi:10.1016/j.dib.2025.112139)
Supplement: Supplementary file 1 [file mmc1.zip › Supplementary_material/RNA-seq/fastQC reports/GSE99621/SRR5642433_1_fastqc.html]

SRR5642433\_1.fastq.gz FastQC Report 

FastQC Report

Thu 1 Feb 2024  
SRR5642433\_1.fastq.gz

## Summary

- Basic Statistics
- Per base sequence quality
- Per tile sequence quality
- Per sequence quality scores
- Per base sequence content
- Per sequence GC content
- Per base N content
- Sequence Length Distribution
- Sequence Duplication Levels
- Overrepresented sequences
- Adapter Content

## Basic Statistics

| Measure | Value |
| --- | --- |
| Filename | SRR5642433\_1.fastq.gz |
| File type | Conventional base calls |
| Encoding | Sanger / Illumina 1.9 |
| Total Sequences | 19860105 |
| Sequences flagged as poor quality | 0 |
| Sequence length | 126 |
| %GC | 43 |

## Per base sequence quality

## Per tile sequence quality

## Per sequence quality scores

## Per base sequence content

## Per sequence GC content

## Per base N content

## Sequence Length Distribution

## Sequence Duplication Levels

## Overrepresented sequences

| Sequence | Count | Percentage | Possible Source |
| --- | --- | --- | --- |
| AAGCAGTGGTATCAACGCAGAGTACTTTTTTTTTTTTTTTTTTTTTTTTT | 2015650 | 10.149241406326905 | Clontech SMART CDS Primer II A (100% over 26bp) |
| AAAAAAAAAAAAAAAAAAAAAAAAAAAAAAAAAAAAAAAAAAAAAAAAAA | 55203 | 0.27795925550242556 | No Hit |
| AAGCAGTGGTATCAACGCAGAGTACATGGGAAGCAGTGGTATCAACGCAG | 34436 | 0.1733928395645441 | Clontech SMARTer II A Oligonucleotide (100% over 25bp) |
| AAGCAGTGGTATCAACGCAGAGTACATGGGGAGGCATTGAGGCAGCCAGC | 29836 | 0.15023082707770175 | Clontech SMARTer II A Oligonucleotide (100% over 25bp) |
| AGCAGTGGTATCAACGCAGAGTACTTTTTTTTTTTTTTTTTTTTTTTTTT | 27896 | 0.1404625000723813 | Clontech SMART CDS Primer II A (100% over 25bp) |

## Adapter Content

Produced by FastQC (version 0.11.7)
